# Clinical observation for acupuncture treatment of a small area of hyperplastic scars in young and middle-aged women

**DOI:** 10.1097/MD.0000000000020790

**Published:** 2020-06-26

**Authors:** Ya-Hong Liu, Jun Xiang, Pei-Pei Han, Chun Yang, Yu-Zhen Wang, Wei Wang, Ping-An Zhang

**Affiliations:** The Second Affiliated Hospital of Shaanxi University of Chinese Medicine, Xianyang, Shaanxi, PR China.

**Keywords:** clinical trials, hypertrophic scars, traditional Chinese medicine

## Abstract

**Introduction:**

Hypertrophic scars are a common disease in plastic surgery, which is the reaction of skin connective tissue to trauma beyond the normal range. Although scholars around the world have explored the tissue structure and formation mechanism of HS for decades, they are not satisfactory the result of. No effective treatment has been found. Therefore, the search for safe and effective treatments for HS has always been the focus of medical attention and research. Acupuncture therapy has a definite effect on HS and has unique advantages.

**Methods/design:**

In this study, we will use our own front-to-back clinical research method. We plan to include 120 young and middle-aged female patients who meet the diagnostic criteria for HS. The untreated HS of the enrolled patients will be used as blank controls. The intervention group will be given acupuncture treatment. The assessment of scar area, color, hardness, thickness, itching and pain will be recorded for 30 days of treatment.

**Discussion:**

This trial may provide evidence regarding the clinical effectiveness, safety, and cost-effectiveness of Acupuncture for patients with HS.

**Trial registration:**

ClinicalTrials.gov, ChiCTR2000032624, Registered on 04 May 2020.

## 1 Introduction

Hypertrophic scars (HS) are a common type of cosmetic lesions and are common diseases in the aesthetic department. With the improvement of people's quality of life, there is also increasing concern about HS. HS can occur at any age, but it often occurs between 10 and 35 years old. Young people are susceptible to the disease, especially during adolescence and pregnancy. The incidence of men and women is similar.^[1]^ Previous studies have shown that women have a higher morbidity rate, which may be a bias caused by women who have higher cosmetic requirements and are more likely to go to the clinic than men. The incidence of HS varies greatly among races.^[2]^ The incidence is higher among blacks and people with darker skin. HS can occur in all races. The incidence of black and Mongolian races is relatively high, estimated to be between 4% and 16%, which is 5 to 15 times that of Caucasians. Differences between regions have a significant impact on the incidence of HS.^[3]^ Europeans living in the tropics are more susceptible to illness than Europeans living in the temperate zone. HS occur in the anterior sternum, back, neck, neck, deltoid region, beard area, and earlobe, and are rare in the upper eyelid, penis, scrotum, areola, palms, soles, and cornea.^[4]^ HS brings great difficulty to the physical and mental recovery of patients. The clinical manifestation of this disease is that the scar is obviously higher than the surrounding normal skin, and the local thickening and hardening. The mechanism of scar formation is currently not fully understood. It is generally believed that due to the inflammatory response of the body, fibroblasts and myofibroblasts proliferate and synthesize a large amount of collagen and matrix, which in turn causes collagen metabolism and arrangement abnormalities.^[5]^ Although scholars from all over the world have explored the organizational structure and formation mechanism of HS for decades, there have been no satisfactory results. No effective treatment has been found.

Common treatment methods for HS currently include surgery, drug therapy, laser therapy, radiation therapy, compression therapy, silicone gel membrane, gene therapy and so on.^[6]^ Among the drug treatments, Western medicine treatment mainly targets extracellular matrix-targeted drugs (including glucocorticoids, pyridone, collagenase, etc.), cell-targeted drugs (including 5-FU, bleomycin, retinoic acid, Colchicine, etc.), biological microenvironment targeting drugs (including growth factor regulating drugs, immunomodulators, anti-inflammatory drugs, anti-allergy drugs) and other directions. Western medicine has achieved obvious results in the local treatment of HS, but it still cannot solve the problem of recurrence of purpura.^[7]^ Traditional Chinese medicine (TCM) has a unique understanding of the disease and rich clinical experience. TCM has a unique understanding of HS. TCM believes that HS is due to the deficiency of positive qi after skin trauma, and the toxic poison stagnate on the surface of the skin, which gradually causes the phlegm and congestion of the body to form on the surface, thereby forming scars. And it is related to their own constitution. Some TCM experts hold that this is related to long-term dampness in the body or dampness of the lungs and stomach, coupled with skin trauma, stagnation of blood and blood stasis, and eventually keloids are formed.^[8]^ Therefore, in the treatment of HS, TCM usually starts with the methods of promoting blood circulation, removing blood stasis, tonifying pain, resolving phlegm and dispersing knots, orally combined with external treatment, or supplemented with acupuncture and point injection. TCM has a good clinical effect on the treatment of this disease. TCM has a definite curative effect on prevention and treatment of HS, and has unique advantages. Oral Chinese medicine, external application of Chinese medicine, acupuncture and moxibustion, and integrated Chinese and Western medicine treatment can be selected. Among them, acupuncture combined with oral medication is not only effective in treating the disease, but also has high psychological acceptance. Moreover, it is safe and secure, with little toxic and side effects, and easy to operate. Acupuncture therapy is easy to learn and use, economical and easy to promote. However, there are few research reports on the treatment of this disease by acupuncture. In this study, acupuncture will be used as an intervention to treat HS, and its efficacy will be objectively evaluated. In this study, we will use our own front-to-back clinical research methods to evaluate the clinical efficacy and related indicators of young and middle-aged female patients who meet HS, and then look for an objective and effective treatment of HS. We aim to provide an objective basis for the clinical efficacy of acupuncture treatment of HS.

## 2 Methods/design

### 2.1 Study design and settings

**Figure 1:**
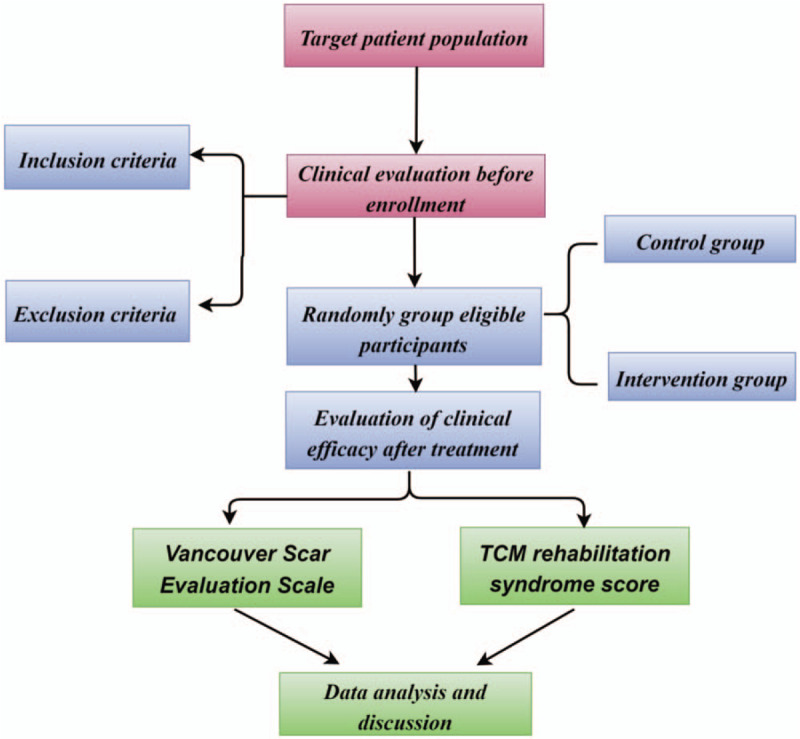
Study design flow chart.

This study will be conducted at the Second Affiliated Hospital of Shaanxi University of Chinese Medicine. This protocol was written and based on Standard Protocol Items: Recommendations for Interventional Trials guidelines. The participants will be informed about the research, procedures, risks, and benefits by YHL (author of this protocol). If they agree, they will sign an informed consent form. Only those participants who read and agree to the protocol and who sign the informed consent form will take part of the study, following the schedule described in Figure 1.

### 2.2 Participants

The subjects of this study will be included in the outpatient and hospitalized patients from the Department of Plastic Surgery of the Second Affiliated Hospital of Shaanxi University of Chinese Medicine, and meet the diagnostic criteria for HS. The research subjects gave informed notification and signed the “*Informed Consent for Pathological Scars Clinical Research*”.

#### 2.2.1 Diagnostic criteria

We will refer to the diagnostic criteria for pathological scars in “*Plastic Surgery*”. We will refer to the “Beauty Surgery” published by the People's Medical Publishing House (Beijing, China). The specific standards are as follows:

(1) HS occurs in various parts of the human body and occurs 4 weeks after trauma.
(2) The lesion is limited to the scope of the wound; the early color is bright red and hard; it often shows excessive keratinization, ulceration and contracture; the texture and bulge of the mature stage are beyond the original damage range, and the mature stage is from June to December.
(3) Consciously itching and burning pain in different degrees.
(4) Symptoms can disappear after several months to 1 to 2 years, and gradually become dark brown, tending to be stable.

In addition, we will refer to the *“Vancouver Scar Scale”* combined with accompanying symptoms to develop scoring criteria, and then grade the severity of HS. The scale has a total score of 21 points. A higher score indicates more severe scarring. Mild: Total points ≤7. Moderate: 7 <total points ≤15. Severe: Total points> 15.

#### 2.2.2 Inclusion criteria

This study will be conducted in China. Patients will be recruited from Plastic surgery departments of the Second Affiliated Hospital of Shaanxi University of Chinese Medicine. We will enroll participants based on the following inclusion criteria:

(1) It meets the diagnostic criteria for HS;
(2) The scar area is less than or equal to 6 cm^2^.
(3) Female patients of 16 ≤ age ≤ 40 years old;
(4) The time for the formation of fatigue marks exceeds 3 months;
(5) Those who have voluntarily signed the informed consent;

#### 2.2.3 Exclusion criteria

Patients will be excluded if they meet the following criteria:

(1) People with allergies and allergies to experimental drugs;
(2) Ages <16 or >40 years;
(3) Patients with renal cancer, bladder tuberculosis, urinary stones;
(4) Patients with other malignant tumors;
(5) Patients with blood system diseases, coagulation dysfunction and autoimmune diseases;
(6) Patients with severe cardiovascular disease, cerebrovascular disease, hematopoietic system disease, and neuropathy;

#### 2.2.4 Conditions for participants to suspend and withdraw from the clinical trial

Researchers participating in clinical trials should carefully record the reasons for the suspension of the trial and the relationship with the clinical trial. It is necessary to clearly record the unwillingness of the subjects to continue the clinical trials, put forward the reasons for withdrawing from the clinical trials, and record the evaluation indicators at the time of discontinuation in detail.

(1) Those who cannot adhere to treatment;
(2) Allergic reactions or serious adverse reactions during the test, the test should be suspended:
(3) Those who have not been treated strictly according to the plan;
(4) People who withdrew from the study on their own.

### 2.3 Interventions

The participants included in the observation will be self-controlled, and each patient randomly selected 3 different scar treatment areas at the same site as observation specimens.

Control group: Take the untreated pathological scars of the participants as blank control. This group will not give any intervention.

Intervention group: Before the treatment, we will carry out strict physical examination and dermatological examination on the patient, and make sure that the patient has no obvious contraindications, and then carry out acupuncture treatment.

**Figure 2:**
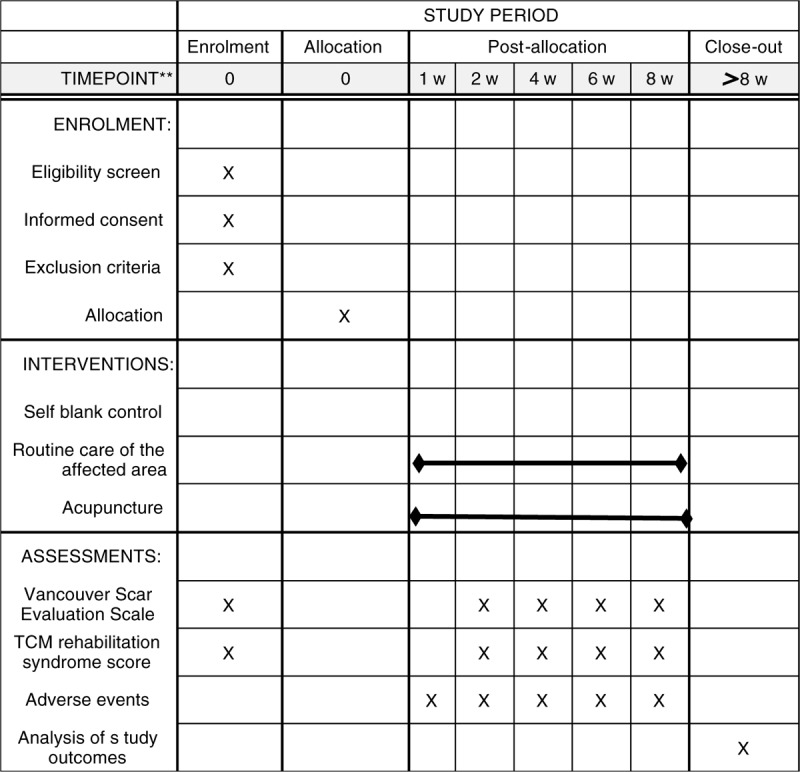
SPIRIT figure for the schedule of enrollment, interventions, and assessments.

(1) Patient position: Choose according to the patient's scar location (supine position, lateral position or sitting position, etc.).
(2) Operation steps: first disinfect the scar area and perform acupuncture in a circular shape on the local scar area of the patient. Needle retention time is 30 min, and the needle is moved twice during the needle retention process. The number of acupuncture depends on the size of the scar area of the affected area, with a minimum of 4 and a maximum of 15, each needle is about 3 cm apart, and the needle insertion is 1 cm to 1.5 cm. At the end of the 30-minute needle retention time, hold the hand on the eye of the needle with a medical swab and proceed in sequence.
(3) Course of treatment: once every two days, 30 days as a course of treatment. Observe the scars every 15 days of treatment (Fig. 2).

Research requirements:

(1) Aseptic operation to prevent infection.
(2) Acupuncture is prohibited when the patient is fasting or fatigued; acupuncture is prohibited in cases of local skin ulcers, tumors, fresh wounds, etc.
(3) Avoid greasy, spicy food, coffee, strong tea and other diets.
(4) Women can’t acupuncture during lactation and pregnancy. People with allergies should use acupuncture with caution.

### 2.4 Outcome measures

Complete the medical records of all patients 1 week before treatment. We will observe the indicators of each group of patients and record the case data on the 15th, 30th, 45th, and 60th day of treatment. All observation records were completed by the same person during the entire treatment period. Therefore, the observation records are all under natural light, room temperature 20 to 24 °C, to keep the room quiet.

#### 2.4.1 Primary outcome measures

We will use the international scar evaluation method: “*Vancouver Scar Evaluation Scale*” as the main standard for evaluating the efficacy.

(1) Color: 3 points for bright red, 2 points for light red, and disappear after pressing, 1 point for non-red or some gray, 0 points for normal skin color;
(2) Height of scar marks: 3 points above 9 mm, 5 mm < 2 points for height ≤9 mm, 1 point for 1–5 mm, 0 points for flat or slightly depressed;
(3) Hardness: 3 points for hard as cartilage, 2 points for hardness like rubber, 1 point for softness, normal for softness Skin score: 0 points;
(4) Itching: 3 points for severe or persistent itching, 2 points for frequent and tolerable itching, sometimes 1 point for itching, and 0 points for no itching;
(5) Pain: very intense 3 points for the “hyperalgesia”, 2 points for moderate-intensity allergic pain, sometimes 1 point for pain, and 0 points for no pain;
(6) tightness: 3 points for strong tightness, not too tight Strong and tolerable score 2 points, slight tightness score, no tightness score 0 points. Efficacy index = (total score before treatment-total score after treatment) / total score before treatment × 100%.

#### 2.4.2 Efficacy evaluation

Refer to “*Guidelines for Clinical Research of New Chinese Medicine*”. Efficacy was evaluated by the ratio of the difference between the points before and after treatment compared to the points before treatment. Syndrome treatment efficiency = (total points before treatment total points after treatment) / total points before treatment × 100%.

(1) Clinical control: clinical symptoms and signs disappeared or basically disappeared, and syndrome scores were reduced by ≥95%;
(2) Significant effect: clinical symptoms and signs were significantly improved, and syndrome scores were reduced by 70%; The signs and symptoms have improved, and the syndrome scores have decreased by ≥30%;
(3) Ineffective: the clinical symptoms and signs have not improved significantly, and the syndrome scores have decreased by less than 30%.

### 2.5 Sample size calculation

The sample size for this trial is based on an expected mean difference between groups of 11 points of the Constant–Murley questionnaire, which is the minimum clinically important difference. To detect this difference between both treatments, with a value of a = 0.05 (probability of committing a type I error) and a statistical power of 95%, a minimum of 49 patients per group is needed. This minimal sample size estimate has been increased by 20% after considering the potential dropouts, finally including 60 patients for each group. Accordingly, the proposed experimental hypothesis is that there will be a difference of at least 11 points in the Constant—Murley questionnaire in the intervention group versus the control group. The sample size was determined using the Stata SE software.

### 2.6 Randomization and blinding

(1) The concealment of random schemes: After the scheme design is completed, the random schemes are enclosed in a sequence-encoded, sealed, light-tight envelope. Then it is handed over to the person in charge of the subject, after screening qualified cases, the envelope is opened to determine the grouping.
(2) The recorder fills in the case report form with basic data and observation of the patient's local and systemic symptoms.
(3) Unified production of various documents.
(4) Check and confirm the correctness and completeness of all research data records and reports and case report forms, and ensure consistency with original data.

### 2.7 Statistical analysis

Data management uses EXCEL software to build a database, double entry, check for outstanding values, and lock. Statistical analysis will be performed using SPSS 25.0 software for statistical analysis. The normality of the measurement data is tested. The data obeying the normal distribution is Student's *t* test, which is expressed by mean ± standard deviation. The data not obeying the normal distribution is rank sum test. And marginal homogeneity test; count data are expressed by rate and composition ratio, and comparison is performed by chi-square test; repeated measurement data are expressed by mean ± standard deviation, intra-group comparison is performed by analysis of variance of repeated measurement data, and inter-group comparison is by multivariate analysis of variance (MANOVA). *P* ≤ .05 indicates that the difference is statistically significant.

### 2.8 Data management

Information obtained from the evaluation of each participant will be recorded on a paper print-out. The information will then be handwritten on a paper document case report form and entered into an Excel file for future statistical analyses. In accordance with the Personal Information Protection Act, the names of all participants will not be disclosed, and a unique identifier number given during the trial will be used to identify participants. All of the participants will be informed that the clinical data obtained in the trial will be stored in a computer and will be handled with confidentiality. The participants’ written consent will be stored by the principal investigator.

### 2.9 Ethics

The study will be conducted under the Declaration of Helsinki principles, as well as following the norms of good clinical practice. Recruitment of patients has not started in this study. The study plan will be submitted to the ethics committee of the Second Affiliated Hospital of Shaanxi University of Chinese Medicine for review. The study protocol will be approved by the ethics committee of the Second Affiliated Hospital of Shaanxi University of Chinese Medicine. The protocol of this study has been registered in the Chinese Clinical Trial Registry with the number ChiCTR2000032624.

## 3 Discussion

HS is a common disease in plastic surgery. It is a manifestation of skin connective tissue response to trauma beyond the normal range. HS often occurs after surgery, trauma and burns, which not only affects the appearance, but also can cause symptoms such as itching, pain, and even cause Severe dysfunction.^[9]^ It is a unique pathological phenomenon after the healing of human skin injury, which brings difficulties to the physical and mental recovery of patients. It is clinically found that the formation of HS is caused by abnormal proliferation of various wound healing processes.^[10]^ However, the formation mechanism of HS is not yet fully understood. It is generally believed that the formation of scars is caused by the imbalance of collagen synthesis and degradation after the body's inflammatory response. The surface layer of HS consists of several layers of epithelial cells to form a very thin and smooth covering layer, and the lower dermis layer is replaced by collagen fibers. Collagen fibers are thick and irregularly arranged, forming an arc-shaped long cord-like collagen to form a vortex-shaped histological scaffold, accompanied by a large amount of fibroblast infiltration.^[11]^ Modern medical scientists have realized through research that when the skin tissue is damaged and damaged to a certain depth, an early inflammatory waterfall-like reaction will appear on the wound surface. Leukocytes, macrophages, mast cells, etc. infiltrate, release a variety of cytokines, fibroblasts and myofibroblasts proliferate and synthesize large amounts of collagen and matrix. This caused the abnormality of collagen metabolism and structural arrangement, coupled with the influence of factors such as microcirculation and oxygen free radicals, which promoted the formation of HS.^[12]^ Sometimes the granulation tissue can be completely absorbed by the surrounding normal tissues after the inflammation factor subsides without going through the scar hyperplasia process, but sometimes it continues to hyperplasia.^[13]^ The development of HS can be divided into three periods, namely hyperplasia, decline and maturity. Scars usually begin to proliferate 1 to 3 months after wound healing, and reach a peak in 6 months. Its clinical features are local hyperemia, bright red color, and active hyperplasia, but it does not invade normal skin. After the proliferative period is the decline period, generally 6 months to 1 year. At this time, the color of the purpura gradually turns to brown, the volume becomes smaller, and the hardness becomes softer. Then enter the mature stage. The specific clinical manifestation is that the scar is obviously higher than the surrounding normal skin, and the local thickening and hardening. In the early stage, due to capillary congestion, the scar surface was red, flushed, or purple. In this period, itching and pain are the main symptoms, and this can even cause the surface to rupture due to scratching. After a certain period of time, the hyperemia of the scar area decreases, the surface color becomes lighter, the scar gradually becomes soft and flat, and the symptoms of itching pain are reduced or disappeared. The length of this hyperplasia period varies with the person and the lesion.

The most common surgical treatment for HS is surgical resection. According to research reports, the rate of HS resection alone is as high as 48% to 100%.^[14]^ However, the effect of this method on wound self-healing is almost zero, so there is still scar formation due to uncontrolled wound healing again. Therefore, surgical treatment should usually be combined with other treatment options, such as steroid injection in the scar, compression therapy, radiation therapy, etc., to prevent recurrence of purpura. In addition, the development of laser technology provides another important auxiliary means for the treatment of purpura.^[15]^ More and more lasers with different wavelengths are being put into use. The thermal effect of the tissue caused by the laser beam depends on the tissue's absorption and conversion of the laser into thermal energy. The high-frequency pulsed laser has a better prospect and is most suitable for the treatment of HS, with an effective rate of more than 57%. Cryotherapy is suitable for smaller scars. Its mechanism of action is to disturb local scar microcirculation through hypothermia, cells are hypoxic and ischemic, leading to cell necrosis, which leads to tissue necrosis and exfoliation ulceration. The effective rate of treatment can reach more than 51%, and the effective rate can be 72% if used in combination with steroid hormones.^[16]^ The side effects of cryotherapy can cause complications such as deepening of local skin pigmentation and mild to moderate skin atrophy. Currently, aesthetic physicians and scientific researchers are actively looking for natural and synthetic antagonists, which affect fibroblasts to produce scar matrix. Therefore, the search for safe, effective and convenient treatments for hypertrophic scars has been the focus of medical research and research in recent years. TCM has a definite curative effect on prevention and treatment of HS, and has unique advantages.^[17]^ Oral Chinese medicine, external application of Chinese medicine, acupuncture and integrated Chinese and Western medicine treatment can be selected. Among them, acupuncture combined with oral medication is not only effective in treating the disease, but also has high psychological acceptance. Moreover, it is safe and secure, with little toxic and side effects, and easy to operate. Acupuncture therapy is easy to learn and use, economical and easy to promote. In this study, we will use our own front-to-back clinical research methods to evaluate the clinical efficacy and related indicators of young and middle-aged female patients who meet HS, and then look for an objective and effective treatment of HS. We aim to provide an objective basis for the clinical efficacy of acupuncture treatment of HS.

## Acknowledgments

The authors would like to thank all the trial participants. The authors are grateful for the support for this study: trial coordinating team, surgical staff, nurses, and research departments.

## Author contributions

YHL, JX, PPH, and YZW designed the study protocol and drafted the manuscript. PAZ reviewed the study protocol and drafted the manuscript. WW and CY are responsible for the statistical design and analysis as trial statistician. All authors carefully read and approved the final version of the manuscript.
